# Developing best practices for PPE Portraits across 25 sites: a systematic assessment of implementation and spread of adaptations using FRAME

**DOI:** 10.1186/s12913-021-06922-2

**Published:** 2021-10-30

**Authors:** Juliana Baratta, Alexis Amano, Paige K Parsons, Stacie Vilendrer, Shira G. Winter, Mae-Richelle Verano, Cynthia Perez, Lucy Kalanithi, Steven M Asch, Mary Beth Heffernan, Cati Brown-Johnson

**Affiliations:** 1 Stanford Division of Primary Care and Population Health, 1265 Welch Rd, CA 94305 Palo Alto, USA; 2 Paige K Parsons Photography, CA 94305 Palo Alto, USA; 3 Occidental College Department of Art and Art History, 1600 Campus Rd, CA 90041 Los Angeles, USA

**Keywords:** PPE Portraits, FRAME, Design thinking, Adaptations

## Abstract

**Background:**

Adaptation, a form of modification that aims to improve an intervention’s acceptability and sustainability in each context, is essential to successful implementation in some settings. Due to the COVID-19 pandemic, clinicians have rapidly adapted how they deliver patient care. *PPE Portraits* are a form of adaptation, whereby health workers affix a postcard size portrait of themselves to the front of their personal protective equipment (PPE) to foster human connection during COVID-19.

**Methods:**

We used the expanded framework for reporting adaptations and modifications to evidence-based interventions (FRAME) method to better understand the reasoning behind and results of each adaptation. We hypothesized that using the FRAME in conjunction with design-thinking would lead to emerging best practices and that we would find adaptation similarities across sites. Throughout multiple implementations across 25 institutions, we piloted, tracked, and analyzed adaptations using FRAME and design thinking. For each adaptation, we assessed the stage of implementation, whether the change was planned, decision makers involved, level of delivery impacted, fidelity to original intervention, and the goal and reasoning for adaptation. We added three crucial components to the FRAME: original purpose of the adaptation, unintended consequences, and alternative adaptations.

**Results:**

When implementing PPE Portraits across settings, from a local assisted living center’s memory unit to a pediatric emergency department, several requests for adaptations arose during early development stages before implementation. Adaptations primarily related to (1) provider convenience and comfort, (2) patient populations, and (3) scale. Providers preferred smaller portraits and rounded (rather than square) laminated edges that could potentially injure a patient. Affixing the portrait with a magnet was rejected given the potential choking hazard the magnetic strip presented for children. Other adaptations, related to ease of dissemination, included slowing the process down during early development and providing buttons, which could be produced easily at scale.

**Conclusions:**

The FRAME was used to curate the reasoning for each adaptation and to inform future dissemination. We look forward to utilizing FRAME including our additions and design thinking, to build out a range of PPE Portrait best practices with accompanying costs and benefits.

**Supplementary Information:**

The online version contains supplementary material available at 10.1186/s12913-021-06922-2.

## Contributions to the literature

- Existing frameworks comprehensively track adaptation characteristics yet lack end-to-end tracking of the adaptation process and inclusion of frontline implementers.
- In the context of COVID-19, rapid adaptation occurred across healthcare settings. For PPE Portraits, we found that iterative prototyping of this intervention led key stakeholders to focus on sustainability during the early development stages of implementation.
- The pragmatic use of both design thinking to integrate end-user feedback during iterative adaptation and FRAME to track adaptations in real time can lead to emerging best practices by creating a bi-direction bridge between theory and on-the-ground implementation.

## Background

Adaptation is a resounding theme of healthcare in 2020. Since the beginning of the COVID-19 pandemic, healthcare organizations underwent radical changes to continue providing high quality care and meet the needs of patients, clinicians, and other key stakeholders. Such adjustments included the widespread adoption of personal protective equipment (PPE) for clinicians and the increased use of virtual care in diverse settings, both implemented worldwide with the purpose of reducing person-to-person transmission of the viral pathogen [1]. These adjustments disrupted human connection, which is central to medical care and patient wellness. Contact isolation has been shown to potentially decrease patient satisfaction and safety [2]. Further, while empathetic care has been associated with improved satisfaction and greater patient empowerment [3], the delivery of such care—facilitated in part through facial expressions and body language—was compromised in patient-clinician interactions due to necessary PPE use and distancing protocols. Navigating these barriers often required novel approaches to care, such as the adaptation of virtual care to the inpatient setting [4]; however, this does not replace the importance of face-to-face communication.

PPE protects clinicians yet poses barriers to patient-clinician communication and connection by masking facial expressions and nonverbal cues [5]. To help restore connection, the PPE Portrait introduces a portraits of healthcare workers themselves on the front of their protective equipment. Anecdotal evidence of PPE Portraits from initial piloting during the Ebola epidemic of 2014 showed improved patient-clinician connection [6]. More recent pilot results demonstrate the perceived benefit of PPE portraits to clinician well-being and to patients in inpatient and outpatient settings including pediatric and adult care [7, 8]. In a world increasingly relying on PPE and universal mask-wearing for pandemic infection control, PPE Portraits have been used across diverse settings, including dozens of hospitals globally, assisted living facilities, and schools. Unsurprisingly, as this intervention was adopted, it was modified to fit a wide range of settings and populations.

Viewed through the lens of implementation science theory, modifications to increase fit to a given set of circumstances are a kind of “adaptation” [9]. Some reasons for adaptations include increased fit for ethno-cultural variation in the target population, promotion of intervention uptake, and sustainability following initial implementation [10, 11]. The nature of modifications can be fidelity-consistent, which keep core elements of the intervention intact, such as changing the length of the intervention, or fidelity-inconsistent, which significantly alter the core intervention, such as diverging from a given intervention protocol entirely [12].

Early modifications by frontline implementers play an important role while best practices and clinical guidelines are being developed. In the clinical setting, such guidelines are critical for replicable, safe, standardized care, yet the development and consistent implementation of such guidelines presents significant challenges [13]. Approaches to creating best practices in the clinical guideline space have been limited to consensus expert opinion such as Delphi panels and nominal groups [14]. These approaches create high-quality best practice guidelines, but the lack of intentional frontline implementer input can create a risk of limited engagement. Therefore, a reverse approach could be to mine on-the-ground pilot implementation (frontline and expert clinician perspectives) for emerging best practices. Design thinking methodologies including iterative feedback from key stakeholders, rapid prototyping, and piloting for feedback have been increasingly used in healthcare to improve an intervention’s acceptability and inform best practices [15]. In the context of an international pandemic, we attempted to describe emerging best practices for using PPE Portraits to foster the patient-clinician connection despite PPE barriers, using both an implementation science-informed and a ground-up approach.

The balance between fidelity and adaptation was systematically tracked through The Framework for Reporting Adaptations and Modifications-Enhanced (FRAME)—a theoretical framework to better understand the process and implications of adaptations. FRAME tracks eight key elements of adaptation: (1) when and how modifications were made; (2) if modifications were planned or unplanned, or proactive versus reactive; (3) who decided to make the modification; (4) the modification itself; (5) the level of delivery at which the modification is made; (6) type of modification (context- or content-level); (7) extent of fidelity of modification to initial innovation; and (8) reason for modification [9]. While other frameworks such as RE-AIM (Reach, Effectiveness, Adoption, Implementation, Maintenance) [16] and MADI (Model for Adaptation Design and Impact) [17] track implementation processes end-to-end including the success of both implementation and intervention outcomes, FRAME highlights key stakeholder influence and is best coupled with the human-centered approach of design thinking by providing a cross-sectional view at a given point in time during the iterative process of adaptation. The developers of FRAME suggest that cultural differences, socio-political factors, and organizational or clinician constraints are all driving reasons for adaptation.

The scientific aim of this work was to use design thinking to implement PPE Portraits and systematically track adaptations across sites using the FRAME. We hoped to achieve the following: (1) assess upstream causes and downstream impacts of adaptations, (2) find cross-cutting adaptation similarities amongst the sites, and (3) expand the FRAME to best capture adaptation nuances. We hypothesized that using designing thinking in conjunction with FRAME would lead to emerging best practices that were both sustainable and acceptable to key stakeholders. We also hypothesized that using the FRAME would help us track adaptations in a structure that could facilitate rapid emergence of best practices.

## Methods

### Participants and Setting

The PPE Portraits as an intervention to improve patient-clinician connection during COVID-19 was first formally documented at Stanford Health Care in March 2020. The pilot evaluation underwent Stanford IRB and received a quality improvement determination (protocol #55,708) [7]. Participating sites were encouraged to submit their own IRB if they planned to collect human subjects data. Otherwise, ethical approval was not needed from each site since involvement was voluntary, with sites contacting Stanford researchers for consultation. Written consent was received from participating sites to be publicly acknowledged in this publication.

Following the Stanford PPE Portrait pilot and subsequent press surrounding this initiative, the quality improvement team fielded requests for advice on how to best implement the intervention. Request for implementation resources and consultations were sent via email and documented by JB. Requesting organizations, which included healthcare and education organizations, assisted living centers, and volunteer services, were situated primarily in the US, but also in Canada, India, Italy, Israel, Japan, and the UK. Furthermore, they served diverse populations in university hospitals, rural community clinics, and US federally qualified health centers (FQHCs). Those on the receiving end of PPE Portraits included pediatric patients, adult patients in inpatient and outpatient settings, assisted living and memory care residents, and school-aged children including preschoolers. PPE Portrait participation recruitment was usually led by a local lead clinician and/or administrator in collaboration with other frontline workers at the implementing organizations (i.e., clinic, schools, assisted living centers).

### Evaluation Team

Qualitive expert Cati-Brown Johnson (CBJ) PhD, along with Juliana Baratta (JB) MS, Paige K. Parsons (PKP) BSAD, and Mary Beth Heffernan (MBH) MFA conducted most consultation meetings. CBJ was the principal investigator (PI) of PPE Portraits at Stanford University with 12 years of mixed-methods research experience; JB was the project manager of the evaluation team with 3 years of qualitive research experience, and MBH was the initial creator of PPE Portraits during the Ebola crisis in 2015 and a co-PI on the evaluation team [18]. Using design-thinking [19], photographer and user experience researcher Paige K. Parsons (PKP), iteratively researched and designed a reusable adaptation based on preferences expressed through email and consultation meetings. PKP facilitated the creation and rollout of PPE Portraits for novel settings (assisted living facilities, schools, hospice organizations). PKP iteratively adapted the portraits based on feedback expressed through email and consultation meetings. CBJ, JB, MBH, and PKP self-identify as women. Other authors contributed to conception, analysis, and writing.

### Best Practice Dissemination

Consultation calls were initiated by the lead clinician or administrator who contacted us via email. The majority of contacts had no previous connection to members on the evaluation team. Implementing organizations discovered PPE Portraits through the following avenues: Stanford School of Medicine PPE Portrait Project webpage [20], national media coverage of the project including The Rachel Maddow Show [21], Jeanne Moos CNN report [22], articles in art magazine Hyperallergic [23] and Smithsonian magazine [24], and through other implementing organizations. Three experts from the interdisciplinary evaluation team (PKP, MBH, CBJ) shared responsibility for conducting half-hour to hour consultation meetings with a lead clinician and/or administrator from the implementing organization over phone or Zoom. Follow-up meetings occurred on an ad hoc basis. We also directed organizations to review our online resources [25] to aid in PPE Portrait creation, including photography tips, guides to creates reusable laminated portraits, and a quick how-to implement page (see Appendix A). Since participating organizations initiated contact with our evaluation team, they also could decline consultation and/or end the rollout of PPE Portraits at their discretion.

During and immediately after consultation meetings, JB, CBJ, and MBH took field notes around implementation questions, requests for PPE Portrait adaptations, and data collection methods. Consultation meetings were not recorded. We reached data saturation with no novel adaptations surfacing near the end of our data collection. The questions from implementing organizations and our team’s answers were collected to inform general best practices, which are publicly available on the PPE Portraits website [25]. Organizations interested in data collection received copies of our quality improvement (QI) study self-reported surveys for their use with staff and clinicians, as well as our template patient feedback sheets and interview protocols. These materials could be used to assess whether PPE Portraits impacted the delivery of care and patient-clinician connection. Administrators and clinicians adapted the surveys to collect data pertinent to their organization and spearheaded any of their own data collection (not reported herein).

### Assessment of Implementation

**Fig. 1:**
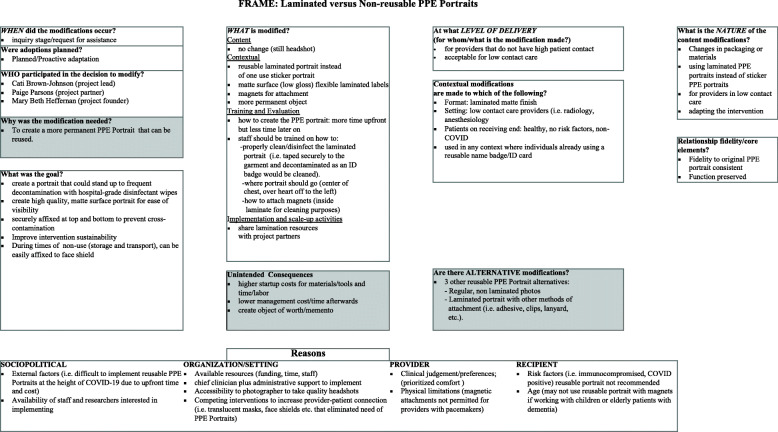
The Framework for Reporting Adaptations and Modifications-Expanded (FRAME). New elements proposed by our team are in the grey shaded boxes: 1) Why was the modification needed, 2) Unintended Consequences, and 3) Are there Alternative modifications?. This modified FRAME covers the adaptation “Laminated versus Non-reusable PPE Portraits.” This figure is our own, created by the authorship team, based on FRAME

From the time of original implementation of PPE Portraits (March 2020), we documented adaptations that supported implementation across a variety of sites and settings. To track partner settings in the process of adapting PPE Portraits, we categorized the implementation process into 4 stages: inquiry stage, early development, late development with imminent rollout, and in practice. At each stage of implementation, we used the expanded Framework for Reporting Adaptations and Modifications to Evidence-based interventions (FRAME) to better understand the reasoning behind and results of each adaptation [9]. For each adaptation, JB used the FRAME’s eight items to assess the implications of each adaptation [9]. The original FRAME, which included definitions and example responses for the 8 systematically tracked components, was available as a reference when recording each adaptation. FRAME modifications proposed by our team included the incorporation of the need addressed by a particular adaptation, any unintended consequences, and alternative adaptations for the same need (Fig. 1). This facilitated end-to-end tracking of need, adaptation, and unintended consequence over time across diverse settings.

In addition to the FRAME, we assessed how adaptations related to the following implementation science outcomes based on Procter, et al.: *acceptability*—satisfaction with various aspects; *appropriateness*—perceived fit; *feasibility*—suitability for everyday use, can be carried out considering resources/training/staff; and *sustainability*—facilitators and barriers to spread [26]. By using the FRAME in conjunction with Proctor outcomes, we simultaneously tracked adaptations made to PPE Portraits’ fit to the target population and assessed how adaptations impacted downstream engagement, *acceptability*, and *sustainability*. Adaptations were then compared to one another to identify emergent themes related to form and function of the adaptations and the adaptation process (i.e., timing, reasoning, key stakeholders, etc.) Other members on the evaluation team (PKP, MV, SV) conducted quality checks of the adaptations including a priori concepts/constructs as well as emergent themes for validity and data-saturation. Our final goal was to describe best practices tailored to site type (hospital, elder care, education) and site circumstances (concerns of cost, set up, patient population, institutional policies, staff members).

## Results

Of the 43 clinicians and administrators who contacted our team for consultation, 25 implemented PPE Portraits at their affiliated organization from March-November 2020 (Table 1). Several requests for adaptations to the portrait format and implementation process arose during early development across these diverse settings. Adaptations were made to meet the needs of clinicians, staff, patients, and beneficiaries, and to adhere to adopting organization’s policies and preferences. Adaptations primarily related to (1) clinician convenience and comfort for *acceptability*, (2) fit to patient populations for *appropriateness*, and to (3) maximize implementation and scaling the intervention for *feasibility* and *sustainability* of PPE Portraits. To support PPE Portrait’s acceptability, adaptations revolved around physical portrait attributes including size, material, and method of affixing the portrait. Adaptations were made to ensure safety and appropriateness for the population on the receiving end of PPE Portraits. Other adaptations focused on implementation and scaling processes: streamlining adoption by hospitals to ensure there was early buy-in from both administrators and clinic staff. Administrators often spearheaded these adaptations, with support from other key stakeholders including clinicians and other care staff. There were pros and cons to each PPE Portrait adaptation.

**Table 1:** Characteristics of the 25 Implementing Sites. This table describes key attributes of the clinic sites including community type, region, site type, affiliation, and size (calculated based on total number of licensed beds including surgical ICU, medical ICU, inpatient care, and emergency care beds.)

Site Characteristic	Sites (*N* = 25)
**Community Type**	
Urban	17 (68%)
Suburban	5 (20%)
Rural	3 (12%)
**Region**	
West	9 (36%)
Northeast	8 (32%)
South	3 (12%)
Midwest	2 (8%)
Canada	2 (8%)
Europe	1 (4%)
**Site Type**	
Hospital	13 (52%)
Intensive care unit	3 (12%)
Pediatric inpatient	3 (12%)
Palliative care	2 (8%)
Outpatient care	3 (12%)
Preschool	1 (4%)
**Affiliation**	
Academic	14 (56%)
Community	7 (28%)
Private	4 (16%)
**Size (licensed beds)**	
30–100	2 (8%)
100–500	6 (24%)
500–1000	7 (28%)
1000+	5 (20%)
Not applicable	5 (20%)

### Clinician convenience and comfort ➜ Acceptability

#### Smaller portrait to enhance staff acceptability

Within a memory unity of an assisted living facility, staff requested modifications to enhance acceptability. The staff were willing to wear a smaller 3 × 4 inch portrait, instead of the recommended 4 × 6 inch portrait, due to anticipated discomfort and self-consciousness associated with wearing the larger portrait. This adaptation was made during early development, and therefore it took less time to modify the portrait before official implementation spread to other staff members.

There were several unintended consequences to this adaptation that impacted initiation, adaptation time, and long-term sustainability. It took a longer time up front to prepare the materials including cutting the 4 × 6 lamination pouch in half. Also, pictures were not available to print in this size, so it required someone familiar with photo manipulation software to specially process the photo files. However, material needs were cut in half, which in the long run reduced resource costs to create the portraits. In addition, the smaller portrait was reportedly physically more comfortable to wear, lighter, and less likely to fall off, which further enhanced staff members’ acceptance of the portrait. After this early adaptation, we continued to recommend 3 × 4 inch portraits at other adopting organizations.

### Patient Needs ➜Appropriateness and Fit

#### Rounded portrait edges, and lanyard use in non-clinical settings

Modifications were made to PPE Portraits to also ensure the safety of patient populations. All modification decisions were made by the portrait wearers for the protection of portrait viewers. In the assisted living center’s memory unit, the square edges of the laminated portrait pouch were perceived to be sharp enough to potentially injure a patient. During early development, staff members requested for the edges to be rounded to remove the risk of poking a patient’s eye or piercing skin.

While magnets were initially used in some settings to affix the portrait, this method of affixation was rejected by preschools, infant centers, as well as assisted living facilities given the potential choking hazard the magnetic strip presented. Alligator clips were used instead and became the primary attachment mechanism. Fidelity to the original intervention was preserved and some teachers used a larger picture portrait so it would be easier to see at a distance. Some staff used lanyards when socially distanced from patients, residents, or students. However, this attachment method was reserved for non-clinical settings due to the risk of cross-contamination caused by the lanyard swinging or flapping and due to the difficulty of lanyard decontamination.

### Alignment with Organization ➜ Feasibility and Sustainability of implementation and scale

#### Facilitative role of onsite champions

PPE Portraits, at most sites, were initiated by a physician, nurse, or administrative champion who contacted our team with inquiries on how to adopt and roll out the intervention. We observed that having a clinician and administrative lead at the site hastened implementation since the priorities of both the health care organization and stakeholders were reflected in the adaptations, leading to long-term acceptability and fewer changes post-implementation. For example, at one hospital, a clinic staff member was concerned about the safety of the portrait affixation method which was escalated to the administrative champion who wrote to our evaluation team: “Currently we are using laminated photos, with clips, and the question has been raised about the possibility of puncturing the gown if using a clip (by one of our ICU nurses). I love this idea of creating a magnet fastener, and if you don’t mind sending the instructions, I will make sure to meet with the person attached to this project on our end. This seems like a great adaptation and look forward to exploring this” (personal communication, June 23, 2020). This adaptation, while voiced by an administrative champion, reflected the needs and concerns of clinic staff. Having the two roles in close communication facilitated delivery and sustainability of PPE Portraits.

Other adaptations related to ease of dissemination included slowing down the process during early development, and providing buttons, which were produced easily at scale but required expensive equipment to produce. Portrait buttons were a novel reusable form, fastened with a safety clip and made from metal or plastic. The adaptation of the button was spearheaded by hospital administrators and clinicians. The pros of the button included secure attachment, ease to put on or take off, and greater societal familiarity with this format, enhancing acceptability from onlookers and adopters. Conversely, the button could be difficult to decontaminate and there was a risk of puncturing PPE; therefore, the button format is recommended in low-risk contamination settings. This portrait modification has limited fidelity to the original intervention since a button is very different from a rectangular portrait- it is circular, and it is seen as less formal, less noticeable, and smaller than a rectangular badge. The button also has wider cultural uses (i.e., sports, politics) where the wearer of the button may not match the subject of the portrait. This can cause confusion when the button is used as a PPE Portrait in the clinical context. However, the button was easier to put on, and did not interfere with clinician workflow, proving more feasible to implement.

#### Encouraging implementation feasibility with onsite photoshoots

The success of PPE Portraits is dependent on receiving clear headshots of healthcare staff members. Coordinating an onsite photoshoot day was the most common and successful model used by clinics. The clinician or administrator champion chose a time when healthcare staff would naturally convene during their busy days including before staff meetings or at the start of a shift, during an organized staff lunch, or during break. Having a point-person to usher staff to the photoshoot location was instrumental in motivating and reminding staff to have their photos taken, especially when the photographer could not enter the building. For clinics where staff were spread over multiple locations, staff would go to the photographer during breaks or time off instead of a coordinated, singular site photoshoot. This allowed staff more flexibility to a choose a time that was convenient for them to have their picture taken. Coordinating within the limits of the clinic organization and remaining flexible were both key to successful photoshoots.

#### Alignment with site needs: single use stickers versus reusable laminated portraits

Between March and May 2020, during the initial phase of the COVID-19 pandemic and early development of PPE Portraits, there was much deliberation on the use of laminated portraits that were reusable versus disposable portrait picture stickers. The portrait picture stickers were the appropriate option in high-risk clinical settings where clinicians would don one-time PPE in order to have direct contact with patients with confirmed or suspected COVID-19 (e.g., ICU, emergency departments). However, the drawbacks of stickers included the need for a volume of stickers, depending on the clinical context. Staff time and resources to replenish these stickers were often not available in settings with competing clinical priorities; leaders observed the practice would be abandoned when stickers ran out.

**Fig. 2:**
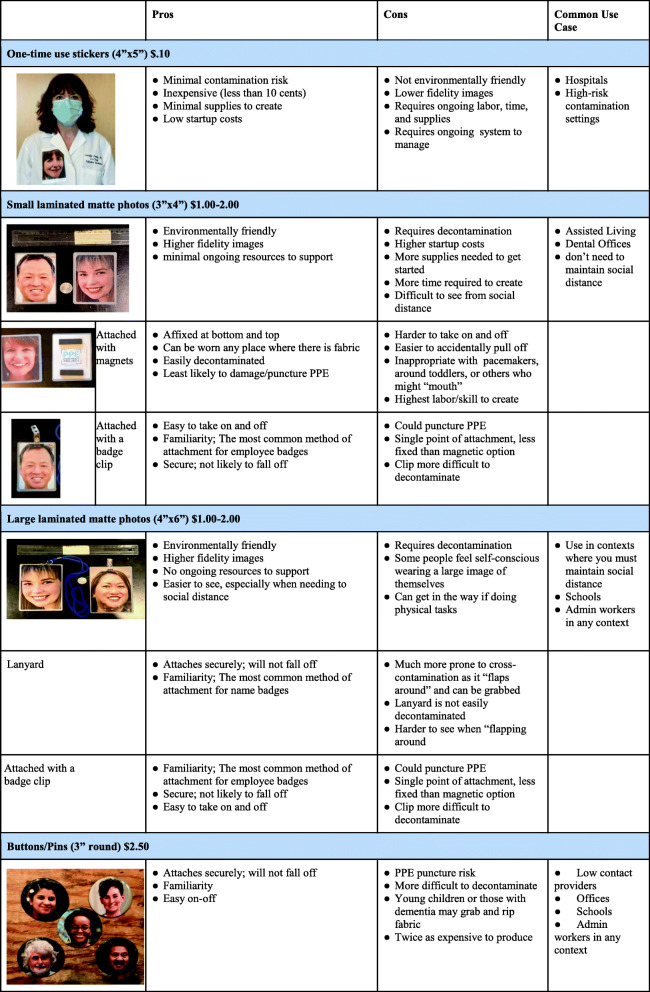
Types of PPE Portraits Pros and Cons outlines the different forms of PPE Portraits (single use, laminated, buttons/pins). This guide is intended for decision makers in organizations to help determine which type of PPE Portrait is most appropriate considering the implementation context, staff, and setting. (Consent was received from all individuals pictured for the use of their PPE Portrait headshot.) This figure is our own, created by authorship team. Written consent for participation has been obtained from the participants shown

Reusable portraits became the most widely used practice for outpatient settings and other low-risk infection settings where direct contact with COVID-19 was not anticipated. Reusable portraits had several pros: environmentally friendly, higher fidelity images, matted/low-gloss surface to improve patient visibility of the portrait, and no ongoing labor/resources to support. Cons to reusable portraits included highest labor/skill to initially create; greater difficulty to take on and off; more supplies, time, and costs to get started; and requiring consistent decontamination (Fig. 2). In pilot testing, reusable portraits tolerated frequent decontamination with alcohol swabs at 70 % Isopropanol, 10 % bleach, or other hospital-grade disinfectant wipes without damage. To prevent cross-contamination, reusable portraits were securely affixed at the top and bottom of the portrait with magnets, which is a puncture-free attachment method. Magnets were also easily decontaminated and were least likely to damage the PPE portrait and clothing. Training was required to ensure proper decontamination of the portrait and affixing to clothing (e.g., placing the magnets underneath the clothing at the top and bottom of the portrait.)

Although widely preferred, the laminated portrait option was not always adopted. Reasons for non-adoption included: hospital policy, lack of clinician or administrative support, up-front time constraints especially during the first few months of COVID-19, and lack of knowledge about virus transmission early on in the pandemic. Fears of cross-contamination and hospital workflow barriers were reasons for non-adoption as expressed by one administrative champion, “My only concern for our ICU wards is that the image must stay in the patient’s room for the day and cannot be taken room to room. So, the removal of the portrait badge would be tricky, if the magnet backing will become loose, under the gown. Regarding using these in other parts of the hospital, this would be completely fine.” While laminated portraits required more time, labor, and resource costs up front, they incurred little to no cost once the portrait was created. In addition, we heard from some sites that the laminated portrait became a memento.

#### Why was the adaptation needed

The driving need for each adaptation was associated with the adaptations’ added value to the portrait from the stakeholder’s perspective. Tracking the justification for an adaptation allowed our team to retrospectively identify whether the solution met the initial need. The specific adaptation need was also closely linked to the nature of the stakeholder requesting the modification, which can inform dissemination to similar organizations. For example, the rounding of portrait edges was initially spurred by staff members concerned about the safety of elderly residents in a memory unit. This form of the portrait is beneficial for the safety of portrait viewers in all implementation contexts. The driving needs for an adaptation are often not visible following modification and implementation, and therefore are important to track to inform future dissemination.

#### Alternative modifications

Previous frameworks did not track alternative modifications, leaving practitioners without a set of modification options, even though multiple modifications may have addressed the same need. For example, to meet the need for a reusable PPE Portrait several modifications arose: non-laminated photo, laminated photo attached with magnets or lanyard, and portrait pins. The systematic consideration and tracking of all potential modification types may help clinicians and administrators identify which adaption will best fit their needs and other stakeholders’ preferences.

#### Unintended consequences

While tracking adaptations using the FRAME, we noticed that every adaptation had unintended consequences. This crucial component was absent from the original FRAME. For example, smaller and laminated (versus non-reusable sticker) portraits reduced material costs. This unintended effect could improve sustainability, but the increased labor up front (i.e. cut and round portrait edges) could serve as a barrier to adoption and widespread dissemination. In addition, the development of laminated, reusable portraits had the unintended consequences of creating meaningful mementos of COVID-19 for healthcare staff members. This addition of unintended consequences to the FRAME helped tie this implementation framework to overall aspects of implementation feasibility and downstream sustainability. Our three additions to the FRAME were tracked side-by-side to assess the relationship between need, goal, and unintended consequences of each adaptation (Table 2).

**Table 2:** Adaptations Tracking: Additions to the FRAME. This abridged version of tracking adaptations includes four new elements that are not included in the FRAME: implementation stage, need for the adaptation, goal of the adaptation, and unintended consequences. Setting and the adaptation itself are also tracked. Emerging adaptations centered around material affixation or organizational-driven adaptations. This figure is our own, created by authorship team

	Implementation Stage	Need	Adaptation	Goal of Adaptation	Setting	Unintended Consequences
Material and Affixation Adaptations	Early Development	Lighter, less noticeable portrait	Smaller portrait	Clinician comfort	Assisted living center	Reduced materials
	Early development	Eliminate sharp portrait edges	Rounded edges	Patient safetyclinician convenience	Assisted living center	Increased up front resources and time
	Imminent rollout	Ease wearing portrait	Button portrait	Scale	Outpatient care	Greater social acceptance; confusion around button’s purpose (i.e. who is the portrait subject)
	Early Development	Long-term use PPE portrait, less on-site process management	Reusable laminated portrait	Scale	Schools, assisted living center	Lower management cost
	Early Development	Reduce risk of cross-contamination	Non-reusable sticker portrait	Patient and clinician safety	Hospital in-patient care	Lower fidelity images
	Implemented/in use	Effective but non-damaging decontaminate	70 % alcoholdecontaminate	Patient and clinician safety	All	Increased frequency of decontamination
**Organizational Adaptations**	Imminent rollout	Quality, high fidelity pictures	Onsite portrait day before staff meeting	Clinician convenience	All	Larger staff buy-in; risk spreading COVID-19 during inside photoshoot so moved to an outside location
	Early Development	Intervention uptake	Administrator plus clinician lead	Scale	Hospital, assisted living center	Few to no adaptations post implementation
	Imminent rollout	Organization recognition	Organization logo in corner of the portrait	Patient trust	Traveling COVID-19 testers	Greater social acceptance; PPE Portrait appeared branded/commercial

## Discussion

**Fig. 3:**
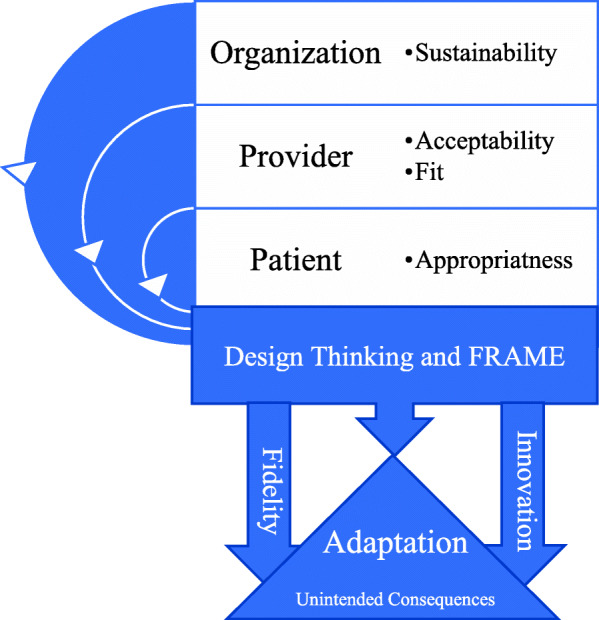
Design Thinking and FRAME were used to track adaptations and balance the competing needs of organizations and the clinician /user of PPE Portraits. While the organization is concerned with sociopolitical factors, sustainability, and feasibility of the intervention, the clinician is concerned with intervention fit to recipient, acceptability amongst other clinicians, and appropriateness considering the setting. With each adaptation, there is a balance between innovation and fidelity to the original intervention. This figure is our own, created by authorship team

While most implementation science research is retrospective, this study is one of the first to develop flexible best practices using implementation science frameworks (the FRAME) and design thinking, demonstrating a bi-directional bridge between implementation theory and practice. We derived adaptation insights from practice using a theory-based framework of adaptation (FRAME) in concert with theoretical implementation outcomes (Proctor) and using those theory-informed insights created practical best-practice guidelines. The FRAME was used not just to document adaptations, but also to drive that information back out to real-world application in the form of tailored best practices (Fig. 3).

The FRAME highlighted how adaptations during rapid implementation were propelled by key stakeholders. This emergent theme of stakeholder-driven adaptations can help us predict the need for future adaptations by adopting organizations. Like most interventions, unique alterations and innovations to PPE Portraits occurred at each new setting, reportedly enhancing implementation, staff uptake, and long-term sustainability.

When making adaptations to meet the needs of key stakeholders, we used design thinking, which prioritizes the needs of the user through a process of consistent user engagement to quickly and iteratively develop multiple prototypes, usually at the start of the adaptation process [15]. We received iterative feedback from implementers through email correspondence and consultation meetings. Design thinking focuses on input from the PPE Portrait users, which in most cases were clinicians, who were also the main advocates of enhancing the intervention’s appropriateness for patients. However, with PPE Portraits, administrators were heavily involved in the decision-making process and may have been less cognizant of the minute-by-minute patient needs due to their distance from direct care. Using design thinking in conjunction with the FRAME helped to balance the often-competing needs of these key stakeholders to create modifications that balanced fidelity to the original invention while fostering the creation of innovative best practices.

### Additions to FRAME

While existing models outline the process adaptations and describe the adaptation itself, the key to each modification is the driving stakeholder. With each local adaptation of PPE Portraits came various modifications to fit stakeholder needs, such as cultural context, organizational limitations, and clinician needs. PPE Portraits experienced rapid dissemination in real-time across institutions, venturing from COVID-19 adaptation, social media posting, single-site pilot at Stanford, and visibility in the press by other implementers independent of our evaluation team. In the best circumstances (i.e., clear administration and clinic lead supporting implementation, clear coordinator with a photographer, and consistent communication with key stakeholders) we have documented implementation taking 3–4 months from initial consultation to rollout.

We used the FRAME to assess the interwoven and often hidden contributing components to each adaptation in order to inform future dissemination to similar settings and track fidelity to the project’s original form. We found the FRAME to be a practical framework for tracking modifications in real time throughout PPE Portraits implementation. However, there is a definite need for an additional “unintended consequences” category to better understand not just what is driving adaptations, but also what their unforeseen impacts are, supporting this bi-directional practice-to-theory-to-practice approach. Also, adding “what is the original need” to the FRAME allowed us to retrospectively assess whether the modification met that need. Finally, the additional tracking of alternative modifications creates a more practical framework that can be used by clinicians and administrators interested in implementing a specific form of PPE Portraits that best fits their specifications based on lessons learned from past iterations.

Overall, the FRAME was helpful in identifying the core elements of PPE Portraits implementation. It highlights the “who” behind adaptations and was therefore best suited to be coupled with design thinking. There are several frameworks for tracking adaptations that did not meet our specific aims. For example, RE-AIM [16] focuses more broadly on the end-to-end implementation process from characterizing and recruiting the target population involved in the initiative (Reach) to tracking use at least 6-months post implementation (Maintenance). MADI framework [17] was also too broad for our purposes since it focuses on the mediators and moderators for the success of both implementation and intervention outcomes. However, MADI or RE-AIM could be used for a future study to retrospectively monitor and evaluate the impact of PPE Portraits on patient and clinician outcomes.

### Main Adaptation Factors

Most adaptations occurred during early development stages or pilot rollout and may have been proactively initiated due to the up-front need for administrative and clinician buy-in before implementation. Though some of the adaptations were planned to accommodate unanticipated preferences post-implementation and during sustainment, the adaptation process was often planned rather than improvised. Adaptations that occurred during sustainment were due to changing demands of the healthcare system. For example, PPE Portraits were initially reusable sticker forms that require less material and organization up front to create. As COVID-19 rates decreased in mid-May 2020 and became better understood, there was greater bandwidth to develop a reusable laminated PPE Portrait and test decontamination methods to ensure the safety of patients and clinicians. In addition to similarities in adaptation process and causes, most adaptations were related to making the portrait reusable or easy and safe to affix to PPE. Several permutations arose to meet these end goals including reusable laminated or button portraits and lanyards or magnets to affix the portrait. Adaptation trends aligned with site type, with hospitals opting for single-use portraits and outpatient settings adopting reusable portrait forms.

When determining if an adaptation is appropriate for future settings, it is important to examine *who* advocated for the modification. For example, modifications made by administrators were often driven by factors such as availability of resources or specific hospital policies. These adaptions were often setting-specific and therefore may have limited external validity. On the other hand, clinician-driven modifications were often developed to meet the needs of the healthcare staff or patients. With clinicians and patients as the common denominator across healthcare settings, the clinician-driven modifications may have greater generalizability to similar healthcare settings.

To improve translational research, there has been a recent push in healthcare to document the implementation and dissemination of interventions. This movement is reflected by the sequential updates to the FRAME over the past 10 years to include additional nuances in the modification process. For example, the framework was expanded to include categories on the process of adaptation in addition to the original categories that simply identified different forms of modifications. The FRAME used for this analysis was an updated version, and we developed a further expanded version to include why the modification was needed, the unintended effects of an adaptation, and alternative adaptations that fit the same need. The FRAME was successfully used to curate the reasoning for each adaptation and to inform future dissemination. We look forward to utilizing FRAME unintended consequences and Proctor outcomes to develop and record a range of PPE Portrait best practices with accompanying costs and benefits.

#### Study Limitations

One limitation for this study is a lack of cost data. Overall cost of reusable versus non-reusable PPE Portraits seemed to vary based on the following factors: type of reusable portrait (i.e., laminated picture, non-laminated picture, portrait button), affixation method (magnets, lanyard, pin), and time plus labor costs. PKP calculated that it would take $682 (in material costs therefore excluding time/labor costs) to create 500 laminated portraits, and another study cited $800 for an initial launch in one unit [8]. Depending on the vendor, non-reusable portrait picture stickers cost on average $75 for 400 standard white matte sticker labels before printing one’s portrait on to the sticker. Another limitation was that we did not differentiate between adaptations and modifications. We used FRAME to track implementation and then identified relevant practices. Therefore, we used the term “adaptations” practically. However, due to the independent nature of this multi-site dissemination, organizations made slight modifications along the way that were not tracked. Also, data was gathered through field notes based on consultation discussions, which may have missed nuances that transcriptions and/or formal interview methods may have gathered. Future researchers using the FRAME should clearly define “modification” and “adaptation” and specify which term best applies to the study at hand.

## Conclusions

The FRAME is an effective tool to move from on-the-ground implementation insights to best practice guidelines that are tailored to site needs and informed by theory and practice. This implementation and diffusion evaluation of PPE Portraits serves as an example of how FRAME can be used to track adaptations in healthcare innovations from their conception through application across diverse environments and populations. We concluded that adaptations are often driven by clinician and institutional preferences and protocols. Therefore, when implementing PPE Portraits or other quality improvement initiatives, it is paramount to consistently include stakeholder feedback, which can be achieved through the iterative process of design thinking and tracked with FRAME. PPE Portraits has the potential to transform the patient-clinician relationship and improve patient experience, especially in situations where widespread use of maximal PPE is required, which can dehumanize the experience of care. As PPE Portraits were implemented in varying settings, the innovation underwent adaptation to meet the needs of the patient population, clinician capacity constraints, organizational environment capabilities, and infection control guidelines. Adaptations improved intervention uptake and promoted sustainability, while in most cases maintaining reasonable fidelity to the initial intervention. These adaptations may streamline future implementation of PPE Portraits at diverse sites. As we move through and beyond pandemic(s), existing and new scenarios of high PPE use (e.g., surgery, autoimmune disorders, other infectious diseases) provide opportunities to continue documenting adaptations through FRAME to inform future PPE Portrait practices.

## Supplementary Information

**Additional file 1:**

## Data Availability

Supporting data is available on request: please contact corresponding author at catibj@stanford.edu.

## Abbreviations

FRAME: Framework for reporting adaptations and modifications-expanded
PPE: Personal protective equipment

## Declarations

### Authors’ Information

The following authors work under the Stanford Division of Primary Care and Population Health: Juliana Baratta MS—social science researcher and project manager, Cati-Brown Johnson PhD—social science research scholar, Alexis Amano MS—social science researcher, Mae-Richelle Verano—clinical research coordinator, Stacie Vilendrer MD, MBA, MS—medical director of the Evaluation Sciences Unit and family physician/instructor, Shira G. Winter, PhD, RN, FNP-BC—postdoctoral research fellow, Cynthia Perez—clinical research coordinator, Lucy Kalanithi MD—clinical associate professor, and Steven Asch MD, MPH—clinical professor. Mary Beth Heffernan MFA is a professor at Occidental College Department of Art and Art History. Paige K. Parsons is a freelance photographer with Paige K. Parsons Photography.
