# Supplementary material for: Developing best practices for PPE Portraits across 25 sites: a systematic assessment of implementation and spread of adaptations using FRAME
Source: BMC Health Serv Res. 2021 Oct 30;21:1182. doi: 10.1186/s12913-021-06922-2 (PMC8556769; doi:10.1186/s12913-021-06922-2)
Supplement: Supplementary file 1 — Additional file 1: [file 12913_2021_6922_MOESM1_ESM.docx]

Appendix A. [**PPE Portraits How-To**](https://www.ppeportrait.org/create/2020/06/20/a-quick-ppe-portrait-how-to/) can be found at <https://www.ppeportrait.org/create/2020/06/20/a-quick-ppe-portrait-how-to/>. It is a quick guide to learning about PPE Portrait creation. This figure is our own, created by authorship team. Written consent for participation has been obtained from the participants shown.
